# Identification of shared gene signatures and molecular mechanisms between chronic kidney disease and ulcerative colitis

**DOI:** 10.3389/fimmu.2023.1078310

**Published:** 2023-02-13

**Authors:** Zhou Liang, Xinrong Hu, Ruoni Lin, Ziwen Tang, Ziyin Ye, Ren Mao, Wei Chen, Yi Zhou

**Affiliations:** ^1^Department of Nephrology, The First Affiliated Hospital, Sun Yat-sen University, Guangzhou, China; ^2^National Health Commission (NHC), Key Laboratory of Clinical Nephrology (SunYat-Sen University) and Guangdong Provincial Key Laboratory of Nephrology, Guangzhou, China; ^3^Guangdong Provincial Key Laboratory of Nephrology, Guangzhou, China; ^4^Department of Pathology, The First Affiliated Hospital, Sun Yat-Sen University, Guangzhou, China; ^5^Department of Gastroenterology, The First Affiliated Hospital of Sun Yat-sen University, Guangzhou, China

**Keywords:** chronic kidney disease, ulcerative colitis, PI3K-Akt signaling pathway, intercellular adhesion molecule 1, neutrophil

## Abstract

**Background:**

There is a complex interaction between chronic kidney disease (CKD) and ulcerative colitis (UC), but the pathophysiological mechanisms underlying the coexistence of CKD and UC are unclear. This study aimed to investigate the key molecules and pathways that may mediate the co-occurrence of CKD and UC through quantitative bioinformatics analysis based on a public RNA-sequencing database.

**Methods:**

The discovery datasets of CKD (GSE66494) and UC (GSE4183), as well as validation datasets of CKD (GSE115857) and UC (GSE10616), were downloaded from the Gene Expression Omnibus (GEO) database. After identifying differentially expressed genes (DEGs) with GEO2R online tool, the Gene Ontology (GO) and Kyoto Encyclopedia of Genes and Genomes (KEGG) pathway enrichment analyses for the DEGs were performed. Next, protein-protein interaction network was constructed with Search Tool for the Retrieval of Interacting Genes (STRING) and visualized by Cytoscape. Gene modules were identified by the plug-in MCODE and hub genes were screened using the plug-in CytoHubba. Then, correlation between immune cell infiltration and hub genes was analyzed, and the receiver operating characteristic curves were used to assess the predictive value of hub genes. Finally, immunostaining of human specimens was used to validate the relevant findings.

**Results:**

A total of 462 common DEGs were identified and selected for further analyses. GO and KEGG enrichment analyses indicated that these DEGs were primarily enriched in immune- and inflammation-related pathways. Among them, the PI3K-Akt signaling pathway ranked top in both discovery and validation cohorts, and the key signal molecule phosphorylated Akt (p-Akt) was shown to be significantly overexpressed in human CKD kidneys and UC colons, and further elevated in CKD-UC comorbidity specimens. Moreover, nine candidate hub genes, including *CXCL8*, *CCL2*, *CD44*, *ICAM1*, *IL1A*, *CXCR2*, *PTPRC*, *ITGAX*, and *CSF3*, were identified, of which *ICAM1* was validated as a common hub gene. Besides, immune infiltration analysis revealed that neutrophils, macrophages, and CD4^+^ T memory cells significantly accumulated in both diseases, and *ICAM1* was remarkably associated with neutrophil infiltration. Furthermore, intercellular adhesion molecule1 (ICAM1)-mediated neutrophil infiltration was validated to be upregulated in kidney and colon biopsies of CKD and UC patients, and further increased in patients diagnosed with both CKD and UC. Finally, ICAM1 had shown critical value as a diagnostic marker for the co-occurrence of CKD and UC.

**Conclusions:**

Our study elucidated that immune response, PI3K-Akt signaling pathway, and ICAM1-mediated neutrophil infiltration might be the common pathogenesis of CKD and UC, and identified ICAM1 as a key potential biomarker and therapeutic target for the comorbidity of these two diseases.

## Introduction

Chronic kidney disease (CKD) refers to a progressive incurable disease characterized by immune dysregulation and multisystem involvement (1). Growing evidence indicates a complex interplay between CKD and gut dysfunction (2–14). Studies revealed that ulcerative colitis (UC), a chronic inflammatory bowel disease affecting the colon and rectum, has a prevalence ranging from 0 to 4.4% in IgA Nephropathy (IgAN, one of the leading causes of CKD), far higher than the highest morbidity of 0.505% in the general population (3, 15). Moreover, IgAN patients with UC displayed more severe renal injury with more proliferation of mesangial cells in renal biopsies (4). Meanwhile, approximately 12% of UC patients suffered from CKD, which is 2.46-fold higher than that of healthy individuals, making CKD one of the most common extraintestinal manifestations (EIMs) of UC (5, 7). Evidently, there exists a strong connection between the occurrence of CKD and UC. However, the mechanisms underlying this phenomenon remain to be explored.

Shared pathological mechanisms, especially immune-mediated inflammation, might explain this clinical observation in CKD and UC. For UC, structural or functional impairment of the intestinal epithelial barrier drives an antigen-activated inflammatory cascade, triggering antigen-presenting cells such as dendritic cells initially (16). As a result, neutrophils are attracted to the inflammatory sites by chemokines and exert pro-inflammatory effects *via* producing IL-23 and neutrophil extracellular traps (NETs) (17). Additionally, immune responses mediated by lymphocytes play crucial roles in UC as well (17, 18). IL-5, IL-6, IL-13, and tumor necrosis factor (TNF) produced by type 2 T helper cells (Th2s), and IL-17 derived from type 17 T helper cells (Th17s) as well as group 3 innate lymphoid cells (ILC3s) can exacerbate colonic barrier dysfunction. Likewise, in CKD, injured tubular epithelial cells secrete inflammatory mediators to recruit immune cells, including macrophages, neutrophils and lymphocytes, which amplify the inflammation and promote renal function failure (19, 20). However, further researches are needed to illustrate the core signaling and associated immunological features in the co-occurrence of these two diseases.

Therefore, our study aimed to investigate the common pathogenic mechanism of CKD and UC, providing insights into both renal EIMs in UC and intestinal immune disorders in CKD patients. Public gene expression database Gene Expression Omnibus (GEO) (http://www.ncbi.nlm.nih.gov/geo/) was employed to identify shared DEGs between CKD and UC for further analyses. We revealed that inflammatory responses and PI3K-Akt signaling pathway were of great importance in both CKD and UC. Besides, we demonstrated that *ICAM1* was a hub gene of DEGs and analyzed its association with immune infiltration. All of the above results were confirmed in validation cohorts and verified in human specimens. Notably, this is the first study to elucidate shared genetic signatures and molecular mechanisms between CKD and UC, which brings a novel angle to the intrinsic link between these two diseases.

## Materials and methods

### Dataset collection

GEO (www.ncbi.nlm.nih.gov/geo/) is a huge public genomics data repository containing gene expression profiles of various diseases. We collected the datasets *via* using ulcerative colitis (UC) and chronic kidney disease (CKD) as keywords for search in the GEO database. The inclusion criteria were as follows: 1) The dataset contained at least fifteen samples in total; 2) The gene expression profiles were from adult human; 3) The included test samples should be from both patients and healthy individuals; 4) Raw data must be provided for further exploration. Finally, GSE66494 (CKD), GSE4183 (UC), GSE115857 (CKD), and GSE10616 (UC) were selected for further analysis (**Table 1**). For datasets GSE4183 and GSE10616, only samples from UC patients were included in the analysis. The GSM numbers of samples used in the analysis were provided in **Supplementary Table 1**.

**Table 1:** Summary of eight GEO datasets involving CKD and UC patients.

No.	GSE number	Platform	Samples	Source type	Disease	Group
1	GSE66494	GPL6480	53 patients and 8 controls	Renal biopsy	CKD	Discovery
2	GSE4183	GPL570	9 patients and 8 controls	Colonic biopsy	UC	Discovery
3	GSE115857	GPL14951	24 patients and 7 controls	Renal biopsy	CKD	Validation
4	GSE10616	GPL5760	10 patients and 11 controls	Colonic biopsy	UC	Validation
5	GSE108112	GPL19983	107 patients and 5 controls	Renal tubulointerstitial compartment biopsy	CKD	Test
6	GSE200818	GPL19983	188 patients and 5 controls	Renal tubulointerstitial compartment biopsy	CKD	Test
7	GSE87466	GPL13158	87 patients and 21 controls	Colon mucosal biopsy	UC	Test
8	GSE47908	GPL570	39 patients and 15 controls	Colon mucosal biopsy	UC	Test

### Identification of common DEGs in both CKD and UC

Differentially expressed genes (DEGs) between diseased group and control group were identified using GEO2R (www.ncbi.nlm.nih.gov/geo/geo2r/) online analysis tool based on R packages (GEOquery and Limma). Genes with p-value < 0.05 and |log2 fold change (log2FC)|>1 were defined as DEGs. The R package “ggplot” was employed to visualize DEGs from datasets using volcano maps. The online Venn diagram tool was used to extract common DEGs both up-regulated or down-regulated between GSE66494 and GSE4183, or GSE115857 and GSE10616 respectively. Additionally, we chose GSE66494 and GSE4183 as our discovery datasets, and GSE115857 and GSE10616 as our validation datasets.

### Functional enrichment analysis

The above shared genes were submitted to the Database for Annotation, Visualization, and Integrated Discovery (DAVID) (https://david.ncifcrf.gov/) online tool for further Gene Ontology (GO) annotation and Kyoto Encyclopedia of Genes and Genomes (KEGG) pathway enrichment analyses. The enriched GO and KEGG pathways with p-value < 0.05 were selected and visualized using bar plots and bubble plots respectively.

### Establishment of protein-protein interaction network

Based on the overlapping DEGs, the Search Tool for the Retrieval of Interacting Genes (STRING) (http://string-db.org/) was employed for protein-protein interaction (PPI). The interaction sources of Textmining, Experiments, Databases, Co−expression, Neighborhood, Gene Fusion and Co−occurrence were adopted. The PPI pairs were extracted with an interaction score > 0.7 and then visualized by Cytoscape 3.9.0. The Cytoscape’s plug-in molecular complex detection (MCODE) was applied to the PPI network to explore key functional modules with selection criteria as follows: K-core = 2, degree cutoff = 2, max depth = 100, and node score cutoff = 0.2. And then, KEGG and GO analyses of the identified modular genes were performed using DAVID online tool and visualized by bubble plots and bar plots respectively.

### Selection and analysis of hub genes

The CytoHubba plug-in of Cytoscape identified hub genes from the PPI network, and then five algorithms (Radiality, MNC {Maximum Neighborhood Component}, MCC {Maximal Clique Centrality}, EPC {Edge Percolated Component}, and Degree) were used to confirm the final hub genes, which were illustrated in Venn diagram. Then, the hub genes were validated in GSE115857 and GSE10616. Ultimately, the above hub genes were submitted to GeneMANIA (http://genemania.org) to construct a co-expression network.

### Receiver operating characteristic curves of hub gene

Receiver Operating Characteristic (ROC) curves were constructed using GraphPad Prism 9, and the area under the ROC curve (AUC) was calculated to assess the predictive value of the hub gene in four testing datasets, including GSE108112 (CKD), GSE200818 (CKD), GSE87466 (UC) and GSE47908 (UC).

### Immune infiltration analysis

To further reveal the immune cell landscape in renal and colonic biopsy, we employed the analytical platform CIBERSORT (https://cibersort.stanford.edu/) and LM22 signature to decode immune cell infiltration profiles of discovery and validation datasets. Pearson correlation analysis was used to identify the relationship between different immune cell phenotypes and hub genes, which was illustrated in lollipop plots.

### Human biopsy specimens

Kidney biopsies in this study were obtained from patients with CKD-UC comorbidity (n=8), patients with CKD without reported comorbid bowel disease (including UC) (n=8) or living donors (n=8). Colon biopsies were derived from patients with CKD-UC (n=8), UC patients without kidney disease (n=8) or normal tissues from colonoscopy (n=8). Samples were obtained from the Department of Nephrology or Department of Pathology, The First Affiliated Hospital of Sun Yat-sen University. All participants provided informed consent and this study was approved by the Ethical Committee of the First Affiliated Hospital of Sun Yat-Sen University.

### Immunofluorescence staining and confocal microscopy

To detect p-Akt, ICAM1 and myeloperoxidase (MPO) in human specimens, slides were processed to remove paraffin and then hydrated in alcohol and phosphate-buffered saline. Then the slides were blocked with 10% normal donkey serum (Sigma, D9663) and incubated with primary antibodies, respectively, at 4°C overnight, including ICAM1 (Santa Cruz, sc-8439, 1:50), p-Akt (CST, 4060S, 1:50), and MPO (abcam, ab208670, 1:150). Later, slides were stained with corresponding fluorescence-labeled secondary antibodies for 1h at room temperature, including anti-rabbit Alexa Fluor 488 (Thermo Fisher, A21206, 1:1000) and anti-mouse Alexa Fluor 546 (Thermo Fisher, A11030, 1:1000). Nuclei were stained with DAPI. Slides were then mounted with Prolong Gold Antifade reagent (Invitrogen, P36934), and examined on a ZEISS LSM880 confocal microscope.

### Statistical analysis

Unpaired t-test was used to detect the difference between two groups. And comparison among more than two groups of immunofluorescence results was assessed by the analysis of variance (ANOVA) using the statistical software GraphPad Prism and P values < 0.05 were considered statistically significant. Statistical significance is defined as * p < 0.05, ** p < 0.01, *** p < 0.001, **** p < 0.0001, or ns (not significant).

## Results

### Identification of common DEGs between CKD and UC

**Figure 1:**
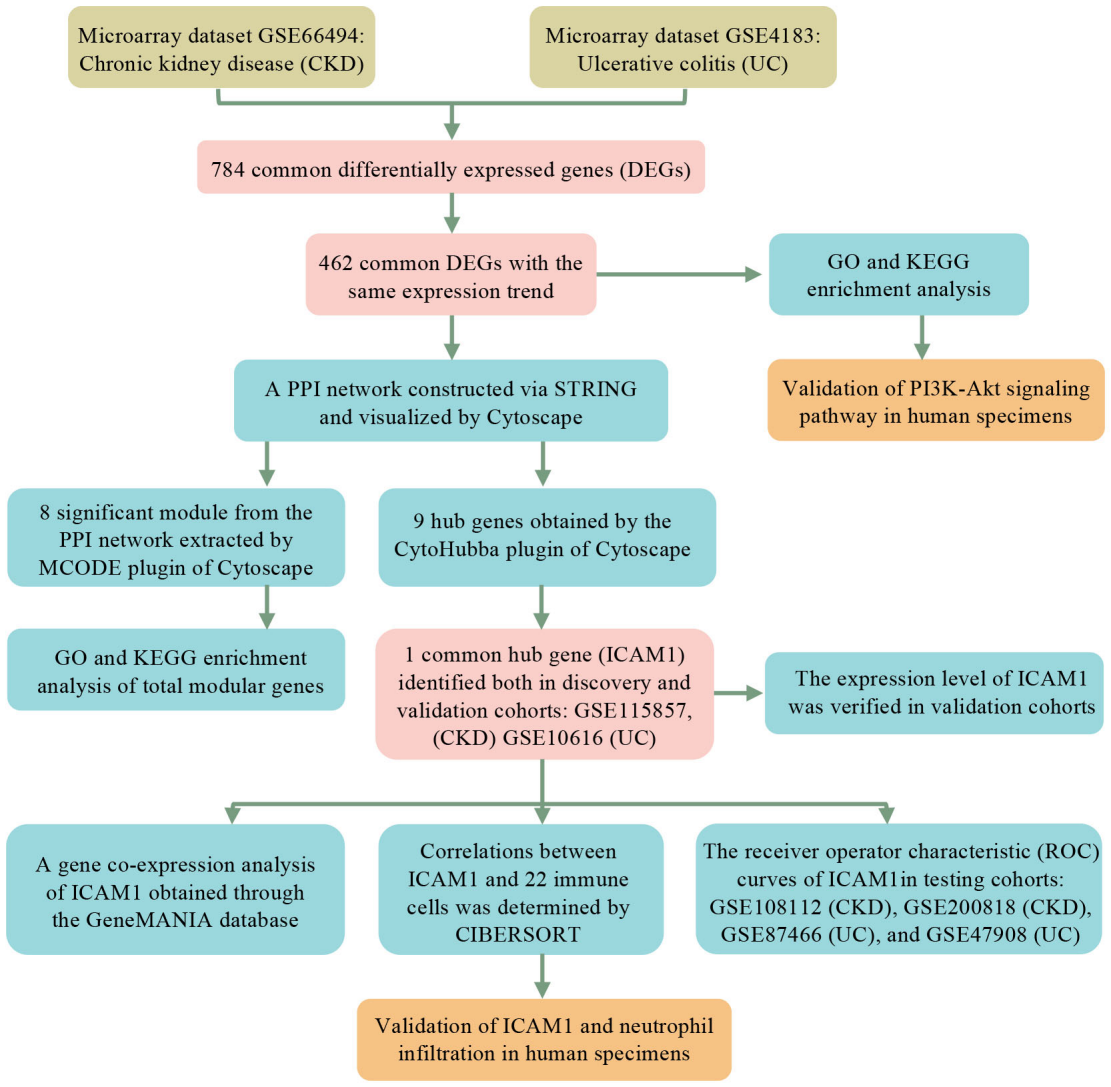
Research design flow chart.

**Figure 2:**
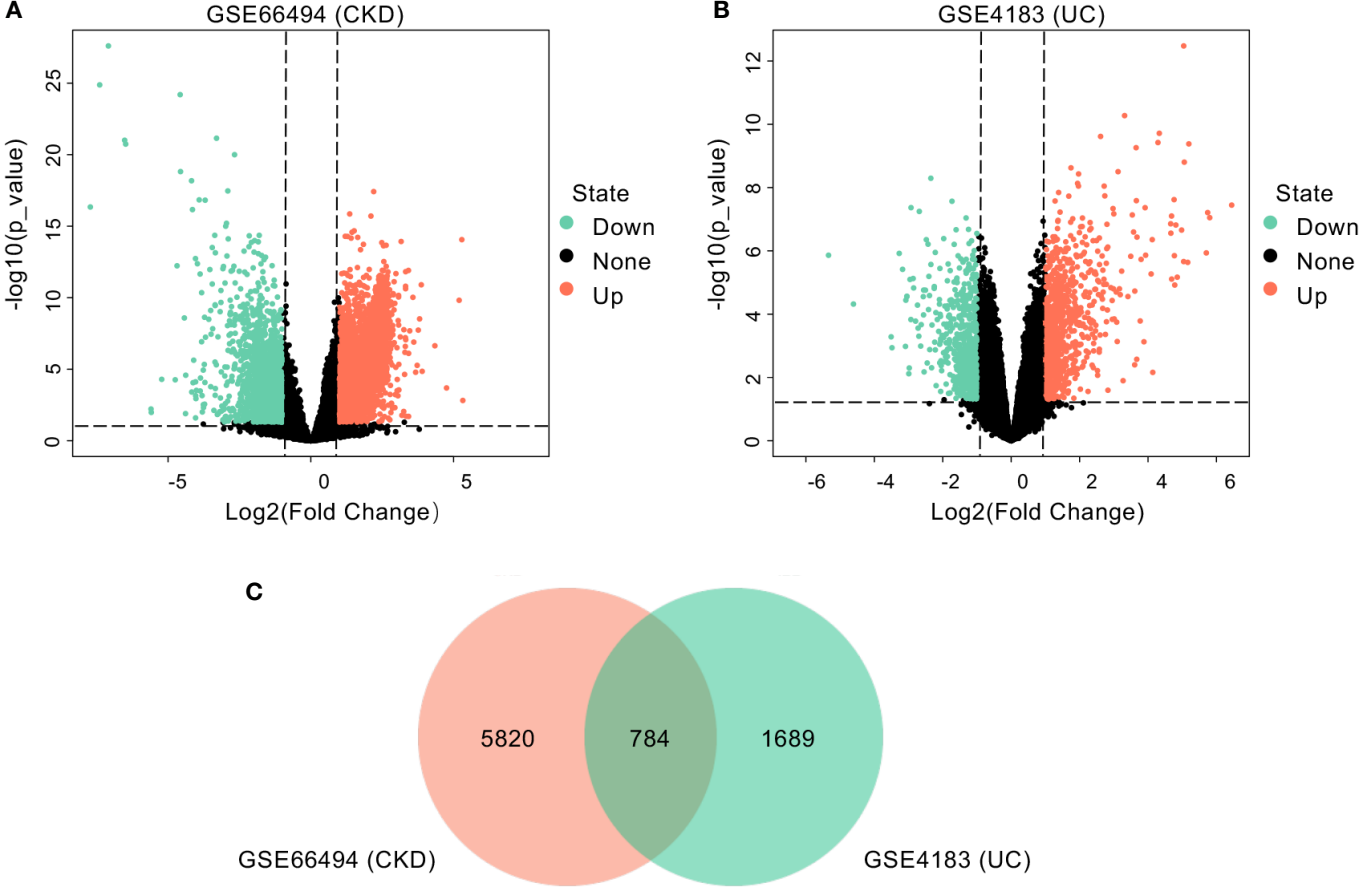
Identification of common DEGs. **(A)** Volcano plot revealed 6604 DEGs between CKD patients and healthy controls. **(B)** Volcano plot revealed 2473 DEGs between UC patients and healthy controls. **(C)** A total of 784 common DEGs were identified after taking the intersection of DEGs in CKD and UC. DEG, differentially expressed gene; CKD, chronic kidney disease; UC, ulcerative colitis.

As shown in the workflow chart summarized in **Figure 1**, CKD microarray dataset GSE66494 and UC microarray dataset GSE4183 were downloaded from the GEO database. Details of selected datasets are provided in **Table 1**. Subsequently, DEGs were identified (6604 in GSE66494 and 2473 in GSE4183) after screening with the criteria of p-value < 0.05 and |log2FC| > 1, which were illustrated by the volcano plots in **Figures 2A, B**, respectively. In addition, a total of 784 shared differentially expressed genes were found after taking the intersection of CKD DEGs and UC DEGs based on Venn diagram analysis (**Figure 2C**). Finally, 462 co-up- or down-regulated DEGs in GSE66494 and GSE4183 were obtained after excluding genes showing opposite expression trends.

### Functional enrichment of common DEGs

**Figure 3:**
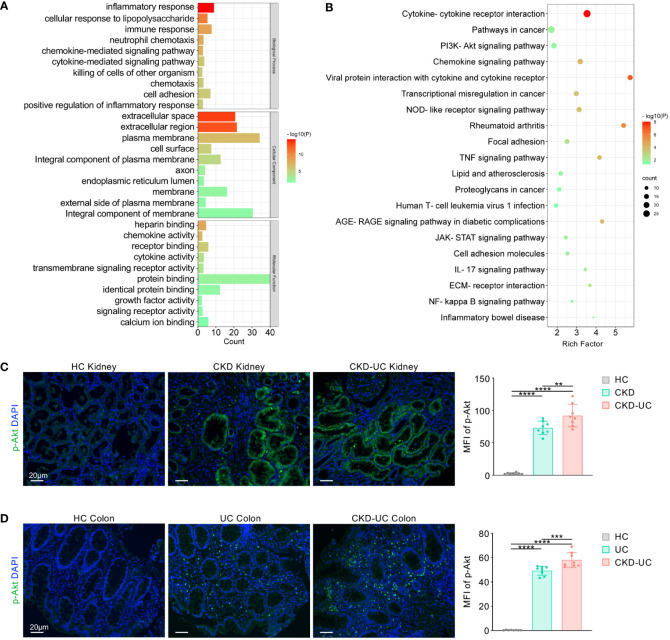
Functional enrichment analysis of common DEGs. **(A)** GO terms in biological process, cellular component, and molecular function were used for functional enrichment clustering analysis on common DEGs. **(B)** KEGG pathway analysis was performed on common DEGs. **(C, D)** Representative immunofluorescence staining images of p-Akt (green) of kidney **(C)** and colon **(D)** biopsies from HC, CKD, UC and CKD-UC patients. Cell nuclei were counterstained with DAPI (blue). Scale bars, 20 μm. The MFI of p-Akt was measured by Image (J) DEG, differentially expressed genes; GO, Gene Ontology; KEGG, Kyoto Encyclopedia of Genes and Genomes; HC, healthy control; CKD, chronic kidney disease; UC, ulcerative colitis; MPO, myeloperoxidase; MFI, mean fluorescence intensity; p-Akt, phospho-Akt. Data in C and D were presented as mean ± SEM, ****P < 0.0001; ***P < 0.0005; **P < 0.005 (ANOVA).

To decipher the biological functions of the common DEGs, GO and KEGG pathway enrichment analyses were performed on 462 shared genes between CKD and UC using the DAVID online tool. For the biological processes of GO enrichment analysis, these genes were primarily enriched in immune-related biological processes, such as inflammatory response, immune response, neutrophil chemotaxis, and chemokine-mediated signaling pathway (**Figure 3A**). For cellular components and molecular functions, these DEGs were enriched in extracellular space, extracellular region, chemokine activity, cytokine activity, heparin binding, and receptor binding. In terms of KEGG pathway enrichment analysis, significantly enriched pathways included cytokine-cytokine receptor interaction, pathways in cancer, PI3K-Akt signaling pathway, chemokine signaling pathway, TNF signaling pathway, IL-17 signaling pathway and NF-kappa B (NFκB) signaling pathway (**Figure 3B**). Taken together, these results indicate that inflammatory response plays a vital role in both CKD and UC, and that extracellular immune mediators such as chemokines and cytokines and PI3K-Akt signaling pathway largely participate in the process.

To confirm our findings, we chose GSE115857 for CKD and GSE10616 for UC as validation datasets, the details of which are presented in **Table 1**. Initially, DEGs with p-value < 0.05 and |log2FC| > 1 were extracted from these two datasets (2001 in GSE115857 and 1450 in GSE10616). Then, Venn diagram analysis was performed on DEGs of the two groups, and a total of 155 common DEGs were spotted (**Supplementary Figure 1A**). Afterward, genes with contrary expression trends were removed, and the remaining 117 DEGs were introduced to GO and KEGG enrichment analyses. For GO enrichment analysis, inflammatory response was enriched, and DEGs were mostly presented in extracellular space as well (**Supplementary Figure 1B**). In terms of KEGG pathway enrichment analysis, PI3K-Akt signaling also ranked top as displayed in **Supplementary Figure 1C**.

Research on PI3K-Akt signaling pathway mainly focused on Akt, which is the most important downstream effector in the PI3K-Akt pathway, and phosphorylation is indispensable for Akt activation (21, 22). Therefore, to validate the PI3K-Akt signaling pathway across disease states, we performed immunofluorescence staining to detect phosphorylated Akt (p-Akt) levels in colon and kidney biopsies from healthy individuals, CKD patients, UC patients, and those suffering from both diseases (CKD-UC). As shown in **Figure 3C**, p-Akt was sparsely expressed in healthy kidney tissues and significantly increased in tubular epithelial cells (TECs) of CKD renal biopsies. Moreover, the expression level of p-Akt was further elevated in CKD patients who also developed UC. An interesting parallel occurred in the colon, where p-Akt, nearly unexpressed in normal human colon, was highly upregulated in colon biopsies from UC patients. A more abundant expression of p-Akt was detected in colon tissues of patients diagnosed with both UC and CKD (**Figure 3D**). To sum all, these findings vigorously indicate that immune regulation and PI3K-Akt signaling pathway might play crucial roles in the occurrence and progression of these two diseases.

### Protein-protein interaction network analysis and submodule analysis

**Figure 4:**
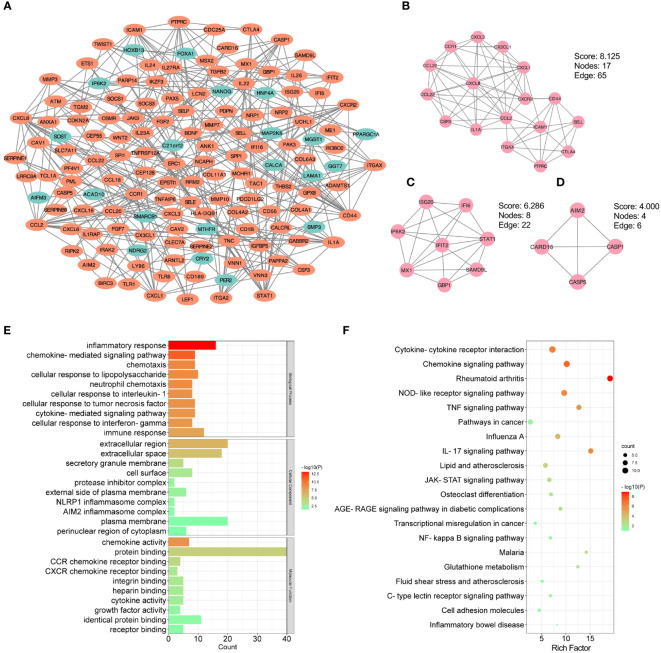
PPI and modular analysis of common DEGs. **(A)** The PPI network of common DEGs. Orange indicates up-regulated genes, and blue-green indicates down-regulated genes. **(B-D)** Top three gene clustering modules in MCODE analysis. **(E, F)** GO and KEGG enrichment analysis of total modular genes. PPI, protein-protein interaction; DEG, differentially expressed genes; GO, Gene Ontology; KEGG, Kyoto Encyclopedia of Genes and Genomes.

In order to further identify the potential relationships between proteins encoded by the shared DEGs of CKD and UC, the PPI network with an interaction score > 0.7 was performed by STRING and visualized with Cytoscape, including 438 nodes and 317 edges (**Figure 4A**). Next, the MCODE plug-in was used to detect significant gene clustering modules. Eight modules consisting of 52 common DEGs and 118 interaction pairs were extracted, and the top three significant modules were shown in **Figures 4B-D**. GO analysis of these modular genes revealed that these modules were mainly enriched in inflammatory response, chemokine-mediated signaling pathway and chemotaxis (**Figure 4E**). KEGG enrichment analysis showed that these modules were highly associated with cytokine-cytokine receptor interaction, chemokine signaling pathway, TNF signaling pathway, and IL-17 signaling pathway (**Figure 4F**). Altogether, these data indicate that inflammation is important in both CKD and UC.

### Identification and co-expression network analysis of hub gene

**Figure 5:**
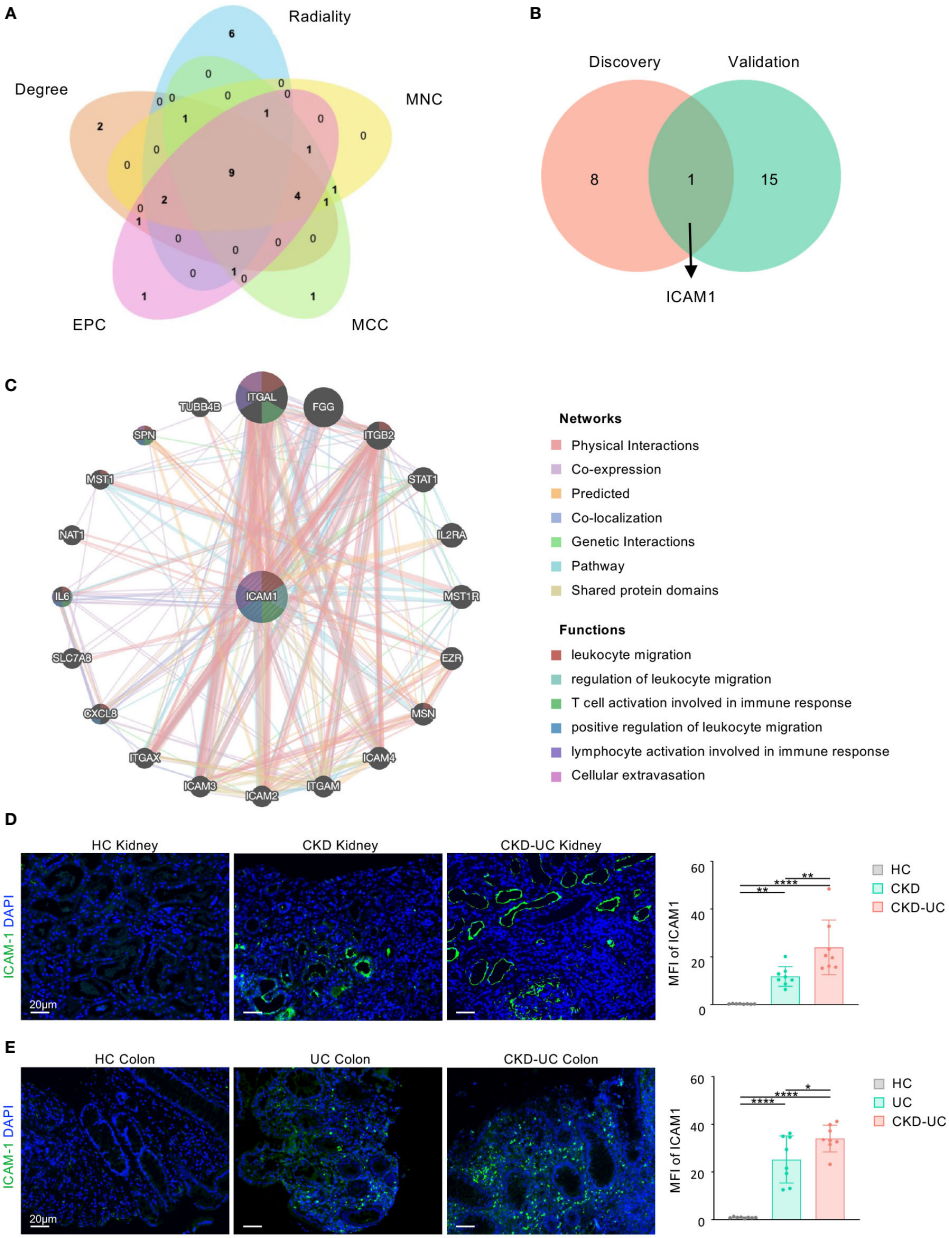
Venn diagram and co-expression network of hub gene. **(A)** The Venn diagram identified 9 candidates for hub genes by five algorithms. **(B)** The Venn diagram of hub genes in discovery cohorts and validation cohorts revealed 1 common hub gene. **(C)** The co-expression genes of *ICAM1* were analyzed *via* GeneMANIA. The 20 most frequently changed neighboring genes are shown. The predicted genes are located in the outer circle, and hub gene is in the inner circle. **(D, E)** Representative immunofluorescence staining images of ICAM1 (green) of kidney **(D)** and colon **(E)** biopsies from HC, UC, CKD-UC patients. Cell nuclei were counterstained with DAPI (blue). Scale bars, 20 μm. The MFI of ICAM1 was measured by Image **(J)** HC, healthy control; CKD, chronic kidney disease; UC, ulcerative colitis; ICAM1, Intercellular cell adhesion molecule-1; MFI, mean fluorescence intensity. Data in **D** and **E** were presented as mean ± SEM, ****P < 0.0001; **P < 0.005; *P < 0.05; (ANOVA).

To investigate the potential genes playing critical roles both in the occurrence of CKD and UC, hub genes of common DEGs were determined by the CytoHubba plug-in. We calculated the scores of common DEGs by five algorithms (Radiality, MNC, MCC, EPC, and Degree) and then took the intersection of the top 20 hub genes obtained from different algorithms by Venn diagram analysis (**Table 2**). Finally, nine candidate hub genes were identified, including *CXCL8*, *CCL2*, *CD44*, *ICAM1*, *IL1A*, *CXCR2*, *PTPRC*, *ITGAX*, and *CSF3* (**Figure 5A**). The full names and related functions of these candidate hub genes are shown in **Table 3**. Interestingly, the majority of these genes were involved in immune cell migration and adhesion, suggesting that the infiltration of inflammatory cells is crucial in the progression of both CKD and UC.

**Table 2:** The top 20 hub genes rank in CytoHubba.

Degree	Radiality	MNC	MCC	EPC
*STAT1*	*CD44*	*CXCL8*	*CCL2*	*CXCL8*
*CXCL8*	*FGF2*	*CCL2*	*CXCL8*	*CCL2*
*CCL2*	*PTPRC*	*ICAM1*	*CXCL1*	*ICAM1*
*CD44*	*CCL2*	*CXCL1*	*CCL20*	*CXCL1*
*ICAM1*	*MMP3*	*CD44*	*CCL22*	*CD44*
*IL1A*	*ICAM1*	*IL1A*	*IL1A*	*IL1A*
*CXCR2*	*CXCL8*	*STAT1*	*CSF3*	*CCL20*
*PTPRC*	*TWIST1*	*PTPRC*	*CXCL3*	*CCR1*
*CXCL1*	*ITGAX*	*CCL20*	*CXCR2*	*CXCR2*
*CCR1*	*IL23A*	*CCR1*	*CCR1*	*PTPRC*
*FGF2*	*NANOG*	*CXCR2*	*ICAM1*	*CCL22*
*ITGAX*	*SELP*	*ITGAX*	*STAT1*	*CXCL3*
*CASP1*	*CSF3*	*CCL22*	*MX1*	*CSF3*
*ITGA2*	*IL1A*	*CXCL3*	*IFIT2*	*ITGAX*
*CCL20*	*CTLA4*	*IFIT2*	*PTPRC*	*FGF2*
*CCL22*	*STAT1*	*CTLA4*	*CD44*	*MMP3*
*MMP3*	*SPP1*	*MMP3*	*ITGAX*	*CTLA4*
*CSF3*	*CXCR2*	*MX1*	*CTLA4*	*SELL*
*SERPINE1*	*SELL*	*CSF3*	*SELL*	*CX3CL1*
*MX1*	*PAX5*	*FGF2*	*IFI6*	*SERPINE1*

**Table 3:** The details of the candidate hub genes.

No.	Gene symbol	Encoded protein	function
1	*CXCL8*	CXCL8, C-X-C motif chemokine ligand 8	A chemotactic factor that mediates inflammatory response by attracting neutrophils and T cells to the site of infection or injury.
2	*CCL2*	CCL2, C-C motif chemokine ligand 2	A chemotactic factor that exhibits a chemotactic activity for monocytes and basophils to participate in inflammatory processes.
3	*CD44*	CD44, CD44 Molecule (Indian Blood Group)	A cell-surface glycoprotein involved in cell-cell interactions, cell, adhesion and migration.
4	*ICAM1*	ICAM1, Intercellular Adhesion Molecule 1	A cell-surface glycoprotein which is mainly expressed on endothelial cells and cells of the immune system and involved in the binding of a cell to another cell or to the extracellular matrix.
5	*IL1A*	IL-1α, Interleukin 1 Alpha	A pleiotropic cytokine produced by monocytes and macrophages involving in various immune responses, inflammatory processes.
6	*CXCR2*	CXCR2, C-X-C Motif Chemokine Receptor 2	This chemokine receptor mediates neutrophil migration to sites of inflammation.
7	*PTPRC*	PTPRC, Protein Tyrosine Phosphatase Receptor Type C	A signaling molecules which is essential in regulating T- and B-cell antigen receptor signaling.
8	*ITGAX*	ITAX, Integrin Alpha X	This protein combines with the beta 2 chain (ITGB2) to form a leukocyte-specific integrin which mediates neutrophils and monocytes adhesion and chemotaxis.
9	*CSF3*	CSF3, Colony Stimulating Factor 3	This cytokine controls the production, differentiation, and function of granulocytes.

We then validated our findings in GSE115857 for CKD and GSE10616 for UC (**Supplementary Figure 2A**). After intersecting the candidate hub genes from discovery cohorts and validation cohorts, we obtained only one shared hub gene, *ICAM1* (**Figure 5B**), which was shown to be significantly upregulated in both CKD and UC of validation cohorts (**Supplementary Figures 2B, C**), suggesting that *ICAM1* may be of great importance in both diseases.

To further verify the reliability and clinical significance of the hub gene discovered by bioinformatics analysis, we obtained the colon and kidney biopsies from healthy individuals, CKD patients, UC patients, and CKD-UC comorbidity patients with the same grouping settings as above, and detected ICAM1 by immunofluorescence. As shown in **Figures 5D, E**, ICAM1 was rarely expressed in healthy kidney and colon tissues, while significantly upregulated in renal TECs and vascular endothelium of CKD patients and colonic mucosae of UC patients, consistent with previous reports (23, 24). Strikingly, the expression levels of ICAM1 were further dramatically elevated in both kidney and colon biopsies from CKD-UC patients, thus confirming the important role of ICAM1 in comorbid features and pathogenic mechanisms. On this basis, we analyzed the co-expression network and related functions of *ICAM1* based on the GeneMANIA database. *ICAM1* possessed a complex interaction network with physical interaction of 77.64%, co-expression of 8.01%, predicted of 5.37%, co-localization of 3.63%, genetic interaction of 2.87%, pathway of 1.83%, shared protein domains of 0.60% (**Figure 5C**). Twenty genes associated with *ICAM1* were identified, mainly related to leukocyte migration, regulation of leukocyte migration, T cell activation involved in immune response, positive regulation of leukocyte migration, lymphocyte activation involved in immune response, and cellular extravasation. These results re-emphasize the importance of immune cell-related inflammation in these two diseases, and *ICAM1* might be the key regulatory gene.

### Association between the hub gene and immune infiltration

**Figure 6:**
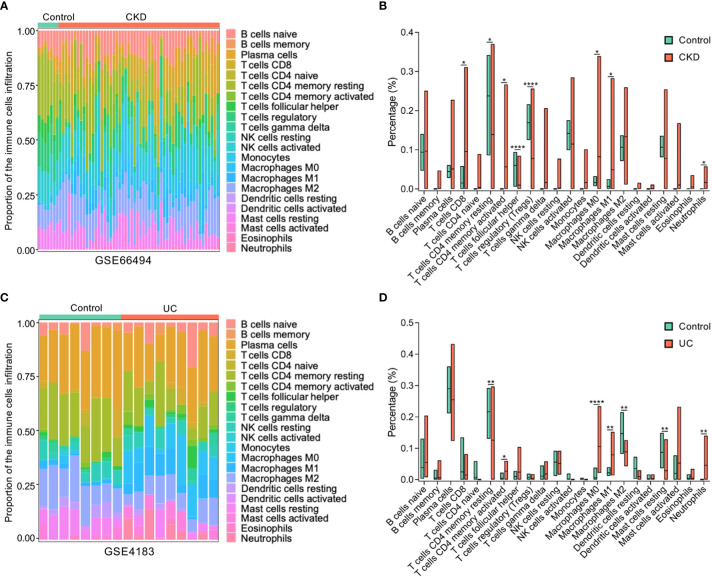
Composition of infiltrating immune cells in CKD and UC. **(A)** The proportion of immune cell populations in kidney was determined by CIBERSORT. **(B)** Comparison of renal immune infiltration between healthy control and CKD patients. **(C)** The proportion of immune cell populations in colon was determined by CIBERSORT. **(D)** Comparison of colonic infiltrating immune cells between healthy control and UC patients. Stacked bar plots show the relative composition of immune cell subsets in CKD **(A)** and UC **(B)**. CKD, chronic kidney disease; UC, ulcerative colitis; ****P < 0.0001; **P < 0.005; *P < 0.05; (unpaired t-test).

To further determine which types of immune cells in the kidney and colon are related to both CKD and UC, the proportions of 22 types of immune cells within kidney and colon samples were quantified by CIBERSORT (**Figure 6A**). Compared to healthy controls, both CKD and UC were commonly characterized by a remarkable enhancement of neutrophils, M0 macrophages (Mφ0), M1 macrophages (Mφ1), and CD4^+^ T memory cells (**Figures 6A-D**).

**Figure 7:**
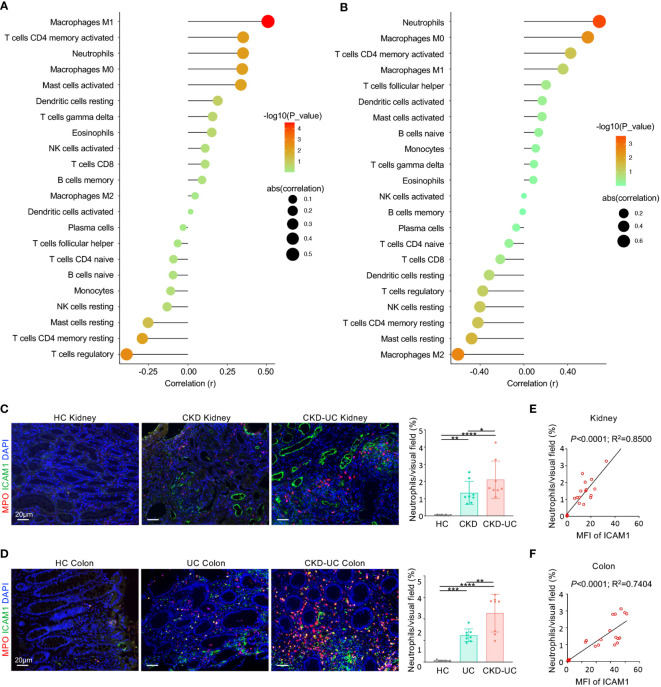
Correlation of hub gene and immune cell infiltration in CKD and UC. **(A, B)** Correlation of ICAM1 expression level and immune cell subtypes in GSE 66494 of CKD **(A)** and GSE4183 of UC **(B)**. **(C, D)** Representative immunofluorescence images of ICAM1 (green) and neutrophil marker MPO (red) of renal **(C)** and colon **(D)** biopsies from HC, CKD, UC, CKD-UC patients. Cell nuclei were counterstained with DAPI (blue). Scale bars, 20 μm. The MFI of ICAM1 was measured by Image J. The quantitative analysis on the percentage of neutrophils per visual field was performed using Image J. **(E, F)** The MFI of ICAM1 was measured by Image J. The relationship between MFI of ICAM1 and neutrophils in renal **(E)** and colon **(F)** biopsies was analyzed with Pearson’s correlation analysis. HC, healthy control; CKD, chronic kidney disease; UC, ulcerative colitis; MPO, myeloperoxidase; MFI, mean fluorescence intensity. Data in C and D were presented as mean ± SEM, ****P < 0.0001; ***P < 0.0005; **P < 0.005; *P < 0.05; (ANONA).

Furthermore, the relationships between these immune cells and *ICAM1* expression levels were analyzed by Pearson correlation analysis. Intriguingly, the expression level of *ICAM1* was positively correlated with neutrophils, Mφ0, Mφ1, CD4^+^ T memory cells, activated mast cells, gamma delta (γδ) T cells, and eosinophils, while negatively linked with regulatory T cells, naive B cells, plasma cells, resting NK cells and resting mast cells in both CKD and UC (**Figures 7A, B**). These matching trends of immune infiltration indicate that the upregulation of *ICAM1* resulted in a similar immune cell landscape in the two diseases. In particular, neutrophils, Mφ0, Mφ1, and activated CD4^+^ T memory cells were the top four significant immune cells positively associated with *ICAM1*, suggesting that *ICAM1* may specifically regulate these four types of immune cells in CKD and UC. The correlation patterns were highly consistent in validated cohorts (**Supplementary Figure 3**).

ICAM1 is a well-known adhesion receptor regulating leukocyte recruitment. In particular, existing evidence suggested that ICAM1 primarily mediates the infiltration of neutrophils in CKD and UC (23, 25). Therefore, we validated the relationship between ICAM1 and myeloperoxidase (MPO) positive neutrophil infiltration in colon and kidney biopsies from healthy controls, CKD patients, UC patients and CKD-UC patients. As expected, ICAM1 expression levels and neutrophil infiltration were both markedly increased in the colon and kidney tissues of UC patients and CKD patients compared with healthy controls, and were further upregulated in CKD-UC patients (**Figures 7C, D**). Most importantly, there was a significant positive correlation between the expression levels of ICAM1 and neutrophil infiltration, which was more pronounced in comorbid patients (**Figure 7E, F**). These results suggest that ICAM1 and subsequent neutrophil infiltration mediated by it might play an important role in the co-occurrence of CKD and UC.

### ROC curves of hub gene

**Figure 8:**
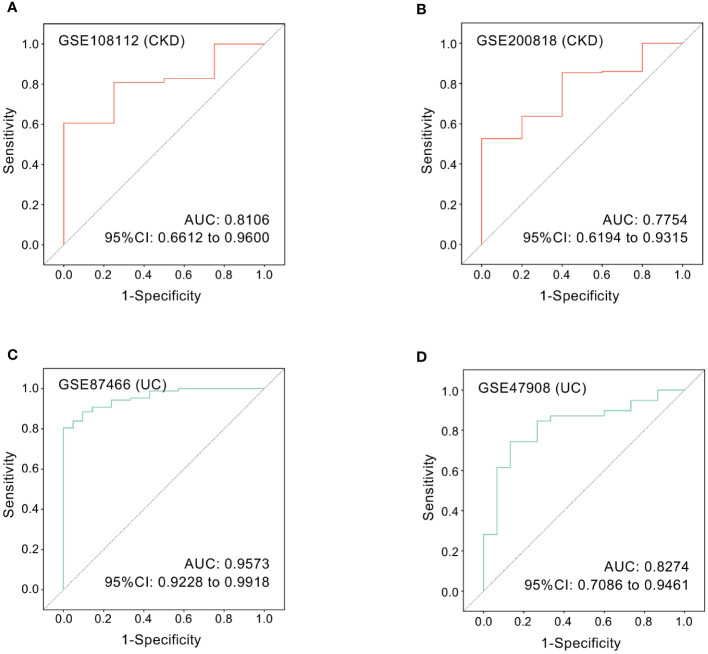
ROC curves of the hub gene in CKD and UC. ROC curves were drawn to evaluate the accuracy of *ICAM1* in diagnosing CKD **(A, B)** or UC **(C, D)**. ROC: receiver operating characteristic; CKD, chronic kidney disease; UC, ulcerative colitis.

Next, we employed ROC curve analysis to explore whether hub gene ICAM1 possesses diagnostic efficiency in the four testing datasets GSE108112 (CKD), GSE200818 (CKD), GSE87466 (UC) and GSE47908 (UC). Details of these datasets are provided in **Table 1**. The AUC values in these four datasets were greater than 0.7, demonstrating that the predictive capabilities of *ICAM1* are excellent. Specifically, in GSE108112 (AUC: 0.8106) and GSE200818 (AUC: 0.7754), *ICAM1* displayed an excellent performance in distinguishing CKD patients from healthy controls (**Figures 8A, B**). Likewise, *ICAM1* worked well to separate UC patients from healthy individuals in GSE87466 (AUC:0.9573) and GSE47908 (AUC:0.8274) (**Figures 8C, D**).

## Discussion

A co-occurrence of CKD and inflammatory bowel disease (IBD), including UC and Crohn’s disease (CD), has long been observed. CKD patients have a higher prevalence of IBD, while renal dysfunction is one of the most common extraintestinal manifestations of IBD (26, 27). Interestingly, it was observed that patients with UC had a significantly higher risk of renal disease than CD patients, suggesting a stronger relationship between UC and CKD. For example, a statistically increased risk for kidney disease was observed in UC but not in CD patients (5, 28, 29). Moreover, CKD patients were more prone to develop UC (prevalence ratio {PR} of 2.46, 95% confidence interval {CI} of 1.40-4.35) than CD (PR, 1.30; 95% CI, 0.75-2.27) (5). In patients with specific etiologies, such as IgAN, a leading cause of CKD, comorbidity of UC was also more common than CD (28). Notably, although multiple etiologies can lead to CKD, including IgAN, diabetic nephropathy, glomerulonephritis, etc., the jury is still out as to which type of CKD is more frequently comorbid with IBD. In IBD patients, the most common renal pathological diagnosis varies across studies (30, 31), with amyloidosis, IgAN and tubulointerstitial nephritis being the most reported. For these reasons, we focused specifically on the link between UC and the onset of CKD, rather than a certain etiological type of CKD. To date, the molecular mechanisms of the connection between CKD and UC remain obscure. In the present study, we sought to investigate the shared gene features and molecular pathways between CKD and UC from a bioinformatics perspective based on the existing sequencing database.

A total of 462 common DEGs with identical expression trends in CKD and UC were identified and subjected to functional enrichment analysis. GO and KEGG analyses revealed that these DEGs were primarily enriched in inflammatory-related pathways, such as inflammatory response, chemokine activity, neutrophil chemotaxis, cytokine-cytokine receptor interaction, TNF and IL-17 signaling pathways. These discoveries underlined the importance of inflammation in the onset and development of CKD and UC. Notably, PI3K-Akt signaling pathway ranked top in both discovery and validation cohorts, suggesting its unique position in the pathogenesis of the two diseases. PI3K-Akt pathway is an intracellular signal transduction pathway involved in multiple biological processes, including metabolism, proliferation, cell survival, growth and angiogenesis (32). The role of PI3K-Akt pathway is well established in cancer, while its importance has also been recognized in various non-cancer diseases (33–35). In CKD, PI3K-Akt signaling pathway mediates oxidized low-density lipoprotein (ox-LDL)-induced nephron loss in podocytes, resulting in renal injury (36). While in UC, inhibition of PI3K-Akt alleviated disruptive epithelial barrier integrity in a mouse model of Dextran Sulfate Sodium (DSS)-induced colitis (33). Additionally, PI3K-Akt pathway also contributes to immune regulation, and is involved in neutrophil chemotaxis and phagocytosis, B cell receptor signaling activation, and dendritic cells maturation (37–39). Here, we observed the expression levels of p-Akt, which represents the activation of PI3K-Akt pathway (22), and found that it was significantly upregulated in kidney tissues of CKD patients and colon tissues of UC patients. Even more impressively, the PI3K-Akt pathway was further activated in kidney and colon biopsies from patients with CKD-UC comorbidity. It is worth emphasizing here that our study obtained both colon and kidney tissue from the same patients with diagnostic comorbidity, and observed identical trends in validated signals within both tissues, which is of particular importance in supporting our proposed pathogenic mechanism for the comorbidity. Taken together, upregulation of the PI3K-Akt pathway might facilitate the progression of immune responses in these two diseases, promoting the co-occurrence of CKD and UC.

Subsequently, according to the CytoHubba plug-in of Cytoscape, we screened nine candidate hub genes, including *CXCL8*, *CCL2*, *CD44*, *ICAM1*, *IL1A*, *CXCR2*, *PTPRC*, *ITGAX*, and *CSF3*, which were all elevated in both CKD and UC patients. Their respective functions are described below. CXCR2, CXCL8 and CCL2 are chemokine receptor and ligands involved in immunoregulatory and inflammatory responses (40). CXCR2 is the receptor for CXCL8, acting as a robust chemotactic factor for neutrophil recruitment and activation (41). CCL2 displays chemotactic activity for monocytes and basophils (42). CD44, ICAM1, and integrin alpha X (ITAX) are adhesion molecules that participate in cell-cell binding and interaction during inflammation or tumor (43–45). IL-1α is a pleiotropic cytokine produced primarily by monocytes and macrophages and is involved in various immune responses (46). Colony stimulating factor 3 (CSF-3) plays major roles in the proliferation, differentiation, and activation of neutrophil cell line hematopoietic cells (47). The identified functions of the above molecules highlighted the role of neutrophils and macrophages in the pathogenesis of CKD and UC. Congruously, taking immune infiltration analysis and histological staining, we revealed that neutrophils and macrophages were significantly accumulated in both CKD and UC biopsies. Collectively, our data indicated that these candidate hub genes regulated the infiltration of neutrophils and macrophages, subsequently resulting in an imbalanced immune response, which played conspicuous roles in both CKD and UC progression.

To further identify the common hub gene, we intersected the candidate hub genes from discovery cohorts and validation cohorts. Ultimately, we found ICAM1 might play an extremely important role in the progression of both CKD and UC. ICAM1 is a cell surface glycoprotein and adhesion receptor primarily expressed by endothelial cells (ECs), epithelial cells and some immune cells (44, 48, 49). The best-characterized function of ICAM1 is to regulate leukocyte recruitment from the circulation to sites of inflammation and mediate their migration across the endothelium (50, 51). Previous studies have demonstrated a pathogenic role of ICAM1 in UC (52, 53). Colonic endothelial cells are by and large ICAM1-negative under the homeostatic state, while are remarkably up-regulated with ICAM1 expression during colitis, consistent with what we discovered here. Targeting ICAM1 was shown to effectively alleviate inflammation, reduce bloody stools and anemia in murine model of colitis. The underlying mechanism was that ICAM1 inhibition prevented the infiltration of pathogenic neutrophils (24). Moreover, clinical trials showed that the antisense oligonucleotide inhibitor of ICAM1, Alicaforsen, was effective and durable in treating UC patients with an excellent safety profile (54). For CKD, ICAM1 overexpression was also detected in human and murine injured kidneys and was found to promote renal dysfunction *via* potentiation of neutrophil-endothelial interactions (23, 55–57). In line with these reports, here we also discovered that *ICAM1* showed a remarkably positive correlation with neutrophils, Mφ0, Mφ1, and activated CD4^+^ T memory cells in both CKD and UC. Given that ICAM1 contribute to the pathogenesis of CKD and UC *via* recruiting neutrophil as discussed above, we further validated the positive correlation between ICAM1 expression levels and neutrophil infiltration in human colon and kidney biopsies. Excitingly, the levels of ICAM1 and neutrophils in the colon and kidney biopsies from patients diagnosed with both CKD and UC were significantly higher than in biopsies from patients with CKD alone or UC alone. Therefore, ICAM1-mediated neutrophil infiltration might play a pivotal role in the pathophysiology of CKD and UC, and patients with a higher level of ICAM1 might be at risk for the two diseases.

It is worth noting that proinflammatory cytokines such as TNFα, IL-1β, and IL-17 were demonstrated to enhance ICAM1 expression *via* NFκB in ECs *ex vivo* (14, 58). While, TNF signaling pathway, IL-17 signaling pathway, and NFκB signaling pathway were significantly enriched in our KEGG analysis of common DEGs, indicating the upregulation of ICAM1 in CKD and UC might be induced by TNFα- and IL-17-mediated NFκB activation *in vivo*. Consequently, the TNFα/IL-17-NFκB-ICAM1-neutrophil pathological pathway might be shared in these two diseases.

However, the present study also has some limitations. First of all, although we have observed the key pathways and hub molecules and immune cells identified in this study on human specimens, further laboratory studies, especially animal experiments, are still needed to verify their pathogenic importance. Secondly, the potential shared mechanisms of CKD and UC were based on common DEGs in patients with CKD or UC, rather than patients with both diseases. As such datasets are not available, validation with datasets including patients with both CKD and UC is not currently possible and should be carried out in the future. At last, we did not explore in the current analysis whether there are shared genetic signatures between CKD and CD, the other major form of IBD. Thus, in order to clarify the specificity between different etiologies, the comorbidity relationship between them will be further investigated in the future.

In conclusion, this bioinformatics study identified shared gene signatures between CKD and UC, illustrating the potential molecular mechanisms of these two diseases. We revealed that an unbalanced immune response, PI3K-Akt signaling pathway, and ICAM1-mediated neutrophil infiltration might be the common pathogenesis of CKD and UC.

## Data availability statement

The original contributions presented in the study are included in the article/**Supplementary Material**. Further inquiries can be directed to the corresponding authors.

## Ethics statement

The studies involving human participants were reviewed and approved by Ethical Committee of the First Affiliated Hospital of Sun Yat-Sen University. The patients/participants provided their written informed consent to participate in this study.

## Author contributions

ZL and XH designed and conducted the entire study and contributed to the bioinformatics analysis. ZL analyzed GEO dataset of UC and XH was in charge of GEO dataset of CKD. RL and ZT performed the laboratory experiments and data analysis. ZY. RM collected human colon biopsies, and WC collected human kidney biopsies. ZL. XH wrote the original manuscript, and WC. YZ revised and finalized the manuscript. All authors contributed to the article and approved the submitted version.
