# Supplementary material for: Identification of shared gene signatures and molecular mechanisms between chronic kidney disease and ulcerative colitis
Source: Front Immunol. 2023 Feb 13;14:1078310. doi: 10.3389/fimmu.2023.1078310 (PMC9970095; doi:10.3389/fimmu.2023.1078310)
Supplement: Supplementary file 1 [file DataSheet_1.pdf]

Supplementary Material

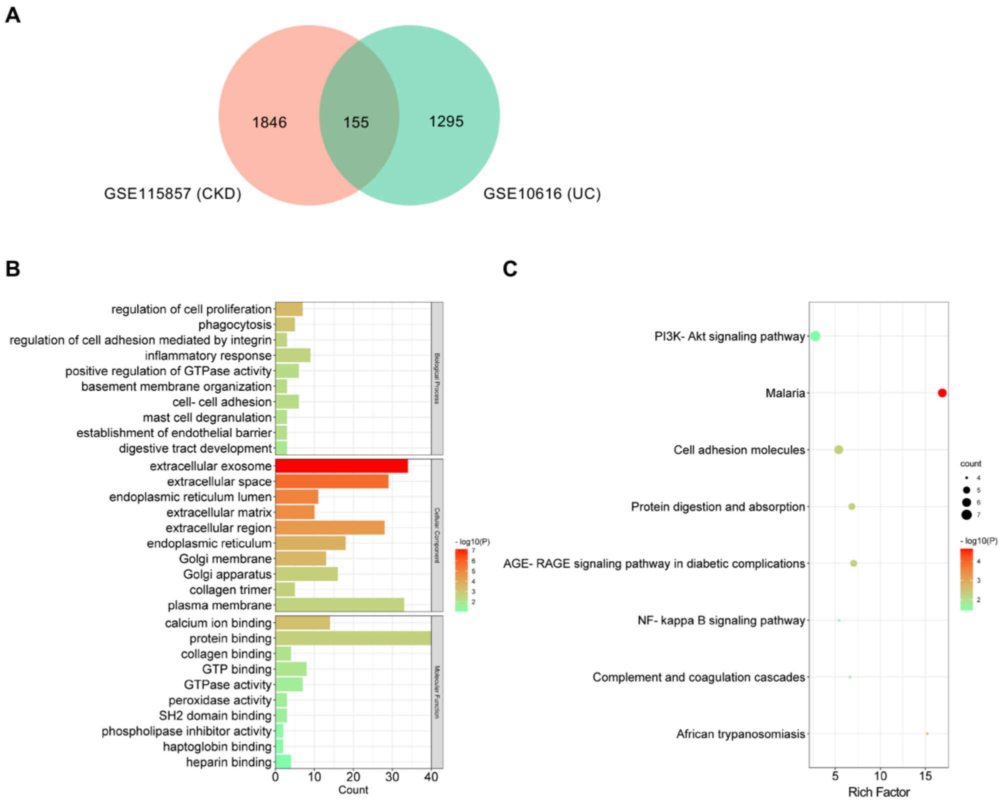

Supplementary Figure 1. Identification and functional enrichment analysis of common DEGs in validation cohorts. (A) The Venn diagram of the common DEGs in CKD and UC of validation cohorts. **(B)** GO terms in biological process, cellular component, and molecular function were used for functional enrichment clustering analysis on common DEGs. **(C)** KEGG pathway analysis was performed on common DEGs. DEG, differentially expressed genes; CKD, chronic kidney disease; UC, ulcerative colitis; GO, Gene Ontology; KEGG, Kyoto Encyclopedia of Genes and Genomes.

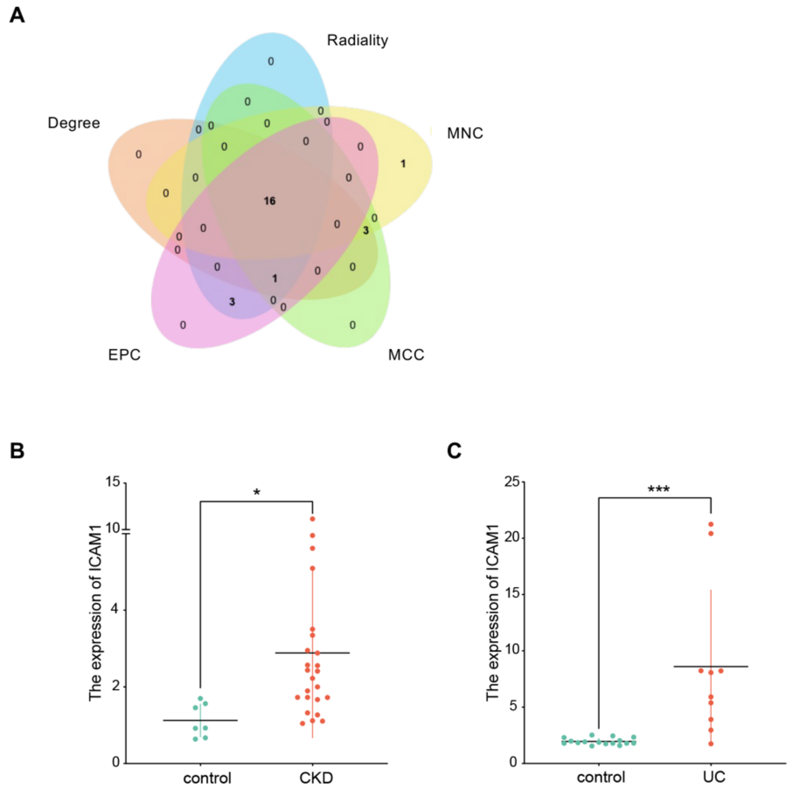

Supplementary Figure 2. Venn diagram and the expression level of hub gene in validation cohorts. (A) The Venn diagram identified 16 candidates for hub genes by five algorithms in validation cohorts. **(B-C)** The expression level of ICAM1 was validated in CKD and UC of validation cohorts. CKD, chronic kidney disease; UC, ulcerative colitis. Data in **B** and **C** were presented as mean ± SEM, ***, P < 0.0005; *, P < 0.05 (unpaired t-test).

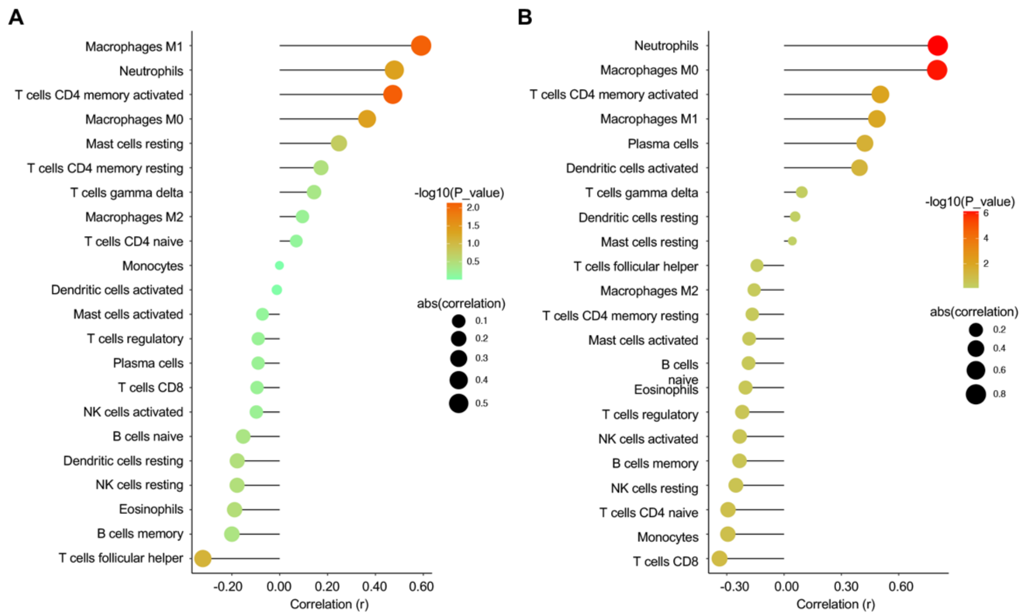

Supplementary Figure 3. Correlation of hub gene and immune cell infiltration in validation cohorts of CKD and UC. (A-B) Correlation of ICAM1 expression level and immune cell subtypes in GSE115857 of CKD **(A)** and GSE10616 of UC **(B)**. CKD, chronic kidney disease; UC, ulcerative colitis.

Supplementary table 1. GSM numbers of each sample in selected datasets.

GSE number	Type	GSM number
GSE66494	HC 8	GSM1623347GSM1623348GSM1623349GSM1623350 GSM1623351GSM1623357GSM1623358GSM1623359
	CKD 53	GSM1623299GSM1623300GSM1623301GSM1623302 GSM1623303GSM1623304GSM1623305GSM1623306 GSM1623307GSM1623308GSM1623309GSM1623310 GSM1623311GSM1623312GSM1623313GSM1623314 GSM1623315GSM1623316GSM1623317GSM1623318 GSM1623319GSM1623320GSM1623321GSM1623322 GSM1623323GSM1623324GSM1623325GSM1623326 GSM1623327GSM1623328GSM1623329GSM1623330 GSM1623331GSM1623332GSM1623333GSM1623334 GSM1623335GSM1623336GSM1623337GSM1623338 GSM1623339GSM1623340GSM1623341GSM1623342 GSM1623343GSM1623344GSM1623345GSM1623346 GSM1623352GSM1623353GSM1623354GSM1623355 GSM1623356
GSE4183	HC 8	GSM95473 GSM95474 GSM95475 GSM95476 GSM95477 GSM95478 GSM95479 GSM95480
	UC 9	GSM95517 GSM95518 GSM95519 GSM95520 GSM95521 GSM95522 GSM95523 GSM95524 GSM95525
GSE115857	HC 7	GSM3191949GSM3191950GSM3191951GSM3191952 GSM3191953GSM3191954GSM3191955
	CKD 24	GSM3191956GSM3191957GSM3191958GSM3191959 GSM3191960GSM3191961GSM3191962GSM3191963 GSM3191964GSM3191965GSM3191966GSM3191967 GSM3191968GSM3191969GSM3191970GSM3191971 GSM3191972GSM3191973GSM3191974GSM3191975 GSM3191976GSM3191977GSM3191978GSM3191893
GSE10616	HC 11	GSM267564 GSM267565 GSM267566 GSM267567 GSM267568 GSM267569 GSM267570 GSM267571 GSM267572 GSM267573 GSM267574
	UC 10	GSM267575 GSM267576 GSM267577 GSM267578 GSM267579 GSM267580 GSM267581 GSM267582 GSM267583 GSM267584
GSE108112	HC 5	GSM2890047GSM2890048GSM2890049GSM2890050 GSM2890051
	CKD 107	GSM2890019GSM2890020GSM2890021GSM2890022 GSM2890023GSM2890024GSM2890025GSM2890026 GSM2890027GSM2890028GSM2890029GSM2890030 GSM2890031GSM2890032GSM2890033GSM2890034 GSM2890035GSM2890036GSM2890037GSM2890038

		GSM2890039GSM2890040GSM2890041GSM2890042 GSM2890043GSM2890044GSM2890045GSM2890046 GSM2890052GSM2890053GSM2890054GSM2890055 GSM2890056GSM2890057GSM2890058GSM2890059 GSM2890060GSM2890061GSM2890062GSM2890063 GSM2890064GSM2890065GSM2890066GSM2890067 GSM2890068GSM2890069GSM2890070GSM2890071 GSM2890072GSM2890073GSM2890074GSM2890075 GSM2890076GSM2890077GSM2890078GSM2890079 GSM2890080GSM2890081GSM2890082GSM2890083 GSM2890084GSM2890085GSM2890086GSM2890087 GSM2890088GSM2890089GSM2890090GSM2890091 GSM2890092GSM2890093GSM2890094GSM2890095 GSM2890096GSM2890097GSM2890098GSM2890099 GSM2890100GSM2890101GSM2890102GSM2890103 GSM2890104GSM2890105GSM2890106GSM2890107 GSM2890108GSM2890109GSM2890110GSM2890111 GSM2890112GSM2890113GSM2890114GSM2890115 GSM2890116GSM2890117GSM2890118GSM2890119 GSM2890120GSM2890121GSM2890122GSM2890123 GSM2890124GSM2890125GSM2890126GSM2890127 GSM2890128GSM2890129GSM2890130
GSE200818	HC 5	GSM6044207GSM6044208GSM6044209GSM6044210 GSM6044211
	CKD 188	GSM6044111GSM6044112GSM6044113GSM6044114 GSM6044115GSM6044116GSM6044117GSM6044118 GSM6044119GSM6044120GSM6044121GSM6044122 GSM6044123GSM6044124GSM6044125GSM6044126 GSM6044127GSM6044128GSM6044129GSM6044130 GSM6044131GSM6044132GSM6044133GSM6044134 GSM6044135GSM6044136GSM6044137GSM6044138 GSM6044139GSM6044140GSM6044141GSM6044142 GSM6044143GSM6044144GSM6044145GSM6044146 GSM6044147GSM6044148GSM6044149GSM6044150 GSM6044151GSM6044152GSM6044153GSM6044154 GSM6044155GSM6044156GSM6044157GSM6044158 GSM6044159GSM6044160GSM6044161GSM6044162 GSM6044163GSM6044164GSM6044165GSM6044166 GSM6044167GSM6044168GSM6044169GSM6044170 GSM6044171GSM6044172GSM6044173GSM6044174 GSM6044175GSM6044176GSM6044177GSM6044178 GSM6044179GSM6044180GSM6044181GSM6044182 GSM6044183GSM6044184GSM6044185GSM6044186 GSM6044187GSM6044188GSM6044189GSM6044190

		GSM6044191 GSM6044192 GSM6044193 GSM6044194 GSM6044195 GSM6044196 GSM6044197 GSM6044198 GSM6044199 GSM6044200 GSM6044201 GSM6044202 GSM6044203 GSM6044204 GSM6044205 GSM6044206 GSM6044212 GSM6044213 GSM6044214 GSM6044215 GSM6044216 GSM6044217 GSM6044218 GSM6044219 GSM6044220 GSM6044221 GSM6044222 GSM6044223 GSM6044224 GSM6044225 GSM6044226 GSM6044227 GSM6044228 GSM6044229 GSM6044230 GSM6044231 GSM6044232 GSM6044233 GSM6044234 GSM6044235 GSM6044236 GSM6044237 GSM6044238 GSM6044239 GSM6044240 GSM6044241 GSM6044242 GSM6044243 GSM6044244 GSM6044245 GSM6044246 GSM6044247 GSM6044248 GSM6044249 GSM6044250 GSM6044251 GSM6044252 GSM6044253 GSM6044254 GSM6044255 GSM6044256 GSM6044257 GSM6044258 GSM6044259 GSM6044260 GSM6044261 GSM6044262 GSM6044263 GSM6044264 GSM6044265 GSM6044266 GSM6044267 GSM6044268 GSM6044269 GSM6044270 GSM6044271 GSM6044272 GSM6044273 GSM6044274 GSM6044275 GSM6044276 GSM6044277 GSM6044278 GSM6044279 GSM6044280 GSM6044281 GSM6044282 GSM6044283 GSM6044284 GSM6044285 GSM6044286 GSM6044287 GSM6044288 GSM6044289 GSM6044290 GSM6044291 GSM6044292 GSM6044293 GSM6044294 GSM6044295 GSM6044296 GSM6044297 GSM6044298 GSM6044299 GSM6044300 GSM6044301 GSM6044302 GSM6044303
GSE87466	HC 21	GSM2332098 GSM2332099 GSM2332100 GSM2332101 GSM2332102 GSM2332103 GSM2332104 GSM2332105 GSM2332106 GSM2332107 GSM2332108 GSM2332109 GSM2332110 GSM2332111 GSM2332112 GSM2332113 GSM2332114 GSM2332115 GSM2332116 GSM2332117 GSM2332118
	UC 87	GSM2332119 GSM2332120 GSM2332121 GSM2332122 GSM2332123 GSM2332124 GSM2332125 GSM2332126 GSM2332127 GSM2332128 GSM2332129 GSM2332130 GSM2332131 GSM2332132 GSM2332133 GSM2332134 GSM2332135 GSM2332136 GSM2332137 GSM2332138 GSM2332139 GSM2332140 GSM2332141 GSM2332142 GSM2332143 GSM2332144 GSM2332145 GSM2332146 GSM2332147 GSM2332148 GSM2332149 GSM2332150 GSM2332151 GSM2332152 GSM2332153 GSM2332154 GSM2332155 GSM2332156 GSM2332157 GSM2332158 GSM2332159 GSM2332160 GSM2332161 GSM2332162

		GSM2332163 GSM2332164 GSM2332165 GSM2332166 GSM2332167 GSM2332168 GSM2332169 GSM2332170 GSM2332171 GSM2332172 GSM2332173 GSM2332174 GSM2332175 GSM2332176 GSM2332177 GSM2332178 GSM2332179 GSM2332180 GSM2332181 GSM2332182 GSM2332183 GSM2332184 GSM2332185 GSM2332186 GSM2332187 GSM2332188 GSM2332189 GSM2332190 GSM2332191 GSM2332192 GSM2332193 GSM2332194 GSM2332195 GSM2332196 GSM2332197 GSM2332198 GSM2332199 GSM2332200 GSM2332201 GSM2332202 GSM2332203 GSM2332204 GSM2332205
GSE47908	HC 15	GSM1162227 GSM1162228 GSM1162229 GSM1162230 GSM1162231 GSM1162232 GSM1162233 GSM1162234 GSM1162235 GSM1162236 GSM1162237 GSM1162238 GSM1162239 GSM1162240 GSM1162241
	UC 39	GSM1162248 GSM1162249 GSM1162250 GSM1162251 GSM1162252 GSM1162253 GSM1162254 GSM1162255 GSM1162256 GSM1162257 GSM1162258 GSM1162259 GSM1162260 GSM1162261 GSM1162262 GSM1162263 GSM1162264 GSM1162265 GSM1162266 GSM1162267 GSM1162268 GSM1162269 GSM1162270 GSM1162271 GSM1162272 GSM1162273 GSM1162274 GSM1162275 GSM1162276 GSM1162277 GSM1162278 GSM1162279 GSM1162280 GSM1162281 GSM1162282 GSM1162283 GSM1162284 GSM1162285 GSM1162286
